# The Central Poor-Law Conference

**Published:** 1914-02-28

**Authors:** 


### THE CENTRAL POOR-LAW CONFERENCE.

### Boards of Guardians and the Insurance Act.

The most interesting feature of the second day's proceedings of the Central Poor-Law Conference at the Guildhall on Wednesday of last week was the paper read by Mr. R. A. Leach, Clerk to the Rochdale Guardians, on the overlapping of old-age pensions, National Insurance, and the Poor Law. Lord Methuen again presided.

Mr. Leach said that according to the last published returns on March 28, 1913, there were 245,418 men and 423,228 women in receipt of the old-age pension of five shillings or under. This number, when compared with the pauper statistics, proved that about four out of every five old-age pensioners were not of the class that, but for the pension, would be compelled to seek ordinary Poor-Law relief. That destroyed the tenableness of the objection sometimes made that the Old Age Pensions Act was simply Poor-Law relief under another name. Mr. Leach said that the overlapping inferred by the title of the paper did not exist; that the one Act and the other and the Poor Law were each separate and distinct in operation, not doing at one and the same time the same things for the same persons. Nevertheless (1) was it fair that the ratepayers should maintain an old-age pensioner in the workhouse infirmary and the old-age pensioner be left to spend his or her pension as he or she might please? (2) Was it fair that the ratepayers should maintain an insured person entitled to any benefit under Part I. of the National Insurance Act, and that person obtain the cash value of the benefit to spend as he or she might please? (3) If it was a benefit that was not payable in cash to the insured person, was it fair that the cash value should not be paid over to the Guardians in relief of the rates? The answer to the three questions was in the negative, and in the interests of the ratepayers amendment of the law was required.

As regards insured persons admitted to the workhouse while entitled to the sickness, disablement, or maternity benefit, all that the Guardians could do at present in the hope of securing some repayment for the maintenance of the insured person without dependent was to declare the relief given to be relief given on loan. But that was far from being enough, and there should be an amendment of the Act so that the Guardians might be able to attach the benefit, in whole or in part, while it remained in the hands of the society or committee administering it. Where they had to provide outdoor medical relief because of failure—be the cause what it might—to administer the medical benefit, or where they had to pay their district medical officer the midwifery fee for attendance in the case where the maternity benefit was payable, or to make good for failure of the sanatorium benefit, it was only right that they should be placed in the statutory position to enforce either against the panel doctor or the approved society or the Insurance Committee a claim up to the Insurance Act benefit for repayment. In the black and white of the Insurance Act was this statutory scandal: Boards of Guardians were not only deliberately excluded from receiving any Parliamentary grant or Treasury assistance towards the institutional provision they made for cases of tuberculosis, but the local Insurance Committees were expressly debarred from making any agreement with them for the reception and maintenance of cases of tuberculosis. The Insurance Act opened wide doors for the administration of Poor-Law relief, but perversely and peevishly barred and locked every door it could against the obtaining of any financial return for it. That it was so was a glaring injustice.

### The Discussion.

In the discussion which followed Mr. Leach's paper various speakers criticised the slowness with which the officials of some of the approved societies under the Insurance Act paid the sickness benefit. It was stated that as a result of that delay many people had had to apply to the Guardians for relief while they were waiting for the insurance benefits to which they were entitled.

Mr. J. Blossom, of Sheffield, declared that in that city there were about two thousand cases every year of insured persons receiving relief from the Guardians. He thought that the ten shillings a week was not enough to maintain a sick man as well as his wife and family.

Mr. C. H. Leach (Darlington) moved a resolution instructing the committee of the Conference to consider the points raised in the paper and to endeavour to act with the Poor-Law Unions Association. The resolution was agreed to.

### Housing and Pauperism.

Miss Constance Cochrane, hon. treasurer of the Rural Housing and Sanitation Association, in a paper on "Rural Housing as it Affects Pauperism," said that one of the most serious defects in a large number of country cottages was the absence of any place in which to keep food except a small dark cupboard in the living-room.

Until village homes were reformed a large portion of the money that was spent on sanatoria might as well be cast into the sea. What chance had a consumptive convalescent when he or she returned to the miserable bedroom with its tiny window, perhaps on a level with the floor, or to the damp ground-floor room with the germ or disease lurking in the cracks and worn-out walls and floor? As a result of the Old Age Pensions Act many more aged persons, especially women, remained in the villages instead of going into the workhouse. A great and serious responsibility rested upon rural district councillors who were also Guardians, for on their right and conscientious administration depended more than upon any other public authority the health and prosperity of the more helpless members of the great human family.

Mr. John S. Nettlefold (Birmingham), chairman of Harborne Tenants, Limited, dealing with the question of housing as affecting pauperism in urban districts, said that rural labourers driven into the towns had to live in a grossly overcrowded slum, because with their wretched wages they could not afford anything better. He was not surprised that many of them ended their lives in the workhouse; the marvel was that they did not all go there. While heredity counted for a good deal, environment had the greater influence on the lives of ordinary people. Suggesting a way out of the present position, Mr. Nettlefold said that they should allow every one who was able and willing to work on the land a fair chance of doing so, and charge him for the land no more than it was now worth. The rating system should be rearranged in such a way that a landowner should not be fined for housing the labourer decently. In urban districts they should open up cheap land for building by the clear-headed and far-sighted administration of the Housing and Town Planning Act, protecting the land from overcrowding and doing away with the so-called "model bye-laws."

The Conference passed a resolution pressing upon the Government the serious condition of the housing problem, and asking that local authorities might be urged to make better use of their existing powers under the Housing and Town Planning Acts.

